# A Giant Baker’s Cyst in a Patient With Rheumatoid Arthritis: An Unusual Case

**DOI:** 10.7759/cureus.99293

**Published:** 2025-12-15

**Authors:** Isabel Monteiro, Rita Sarria, Gisela Moreira Pinheiro, Rui Môço

**Affiliations:** 1 Internal Medicine, Pedro Hispano Hospital, Matosinhos Local Health Unit, Matosinhos, PRT

**Keywords:** baker's cyst, knee pathology, mri- magnetic resonance imaging, popliteal mass, rheumatoid arthritis

## Abstract

Baker’s cysts are fluid-filled swellings in the popliteal fossa commonly associated with degenerative and inflammatory joint diseases, including rheumatoid arthritis (RA), as well as with joint injuries. While they are usually small and asymptomatic, in rare cases, they can enlarge significantly and cause clinically relevant complications.

We describe the case of a 69-year-old man with an undiagnosed RA who presented at his first Internal Medicine consultation with a five-week history of progressive swelling in the left popliteal region extending to the mid-calf, accompanied by pain on movement and difficulty walking. Clinical evaluation revealed a large popliteal mass, and subsequent magnetic resonance imaging (MRI) confirmed a well-defined, heterogeneous cyst measuring 24 cm in its longitudinal axis, causing compression of adjacent structures and exerting a mass effect on the medial gastrocnemius muscle without evidence of rupture.

This case highlights the potential for Baker’s cysts to reach exceptional dimensions, emphasizing the importance of accurate diagnosis for appropriate management and treatment planning.

## Introduction

Baker’s cysts are synovial fluid collections that arise from the gastrocnemio-semimembranosus bursa through a communication with the knee joint [1]. They are frequently associated with intra-articular pathology, particularly osteoarthritis, joint injury and rheumatoid arthritis (RA) [2]. In RA, chronic synovitis and increased intra-articular pressure favor the development of cysts, which are usually small and asymptomatic [3].

The clinical presentation is variable. While many cysts are discovered incidentally during imaging, others manifest with posterior knee pain, swelling, or a palpable mass in the popliteal fossa that becomes more prominent with knee extension or physical activity [4]. Although most Baker’s cysts remain small, giant cysts are rare but clinically significant, as their size and mass effect can complicate the differential diagnosis, overlapping with soft tissue tumors, vascular aneurysms, or thrombosis [5,6]. Larger cysts may also cause stiffness, limited range of motion, or compressive symptoms involving adjacent vascular or neural structures [4]. In rare cases, rupture into the calf may mimic deep vein thrombosis, producing acute pain and swelling, a condition known as “pseudothrombophlebitis syndrome” [7]. Accurate recognition is therefore essential for appropriate management [5,6].

We present the case of an elderly patient with RA who developed an unusually large Baker’s cyst measuring 24 cm, successfully managed with surgical drainage and immunosuppression. This case highlights both the rarity and the clinical implications of giant Baker’s cysts in patients with RA.

## Case presentation

A 69-year-old man was referred to an Internal Medicine clinic with a one-year history of inflammatory polyarthritis involving the metacarpophalangeal and proximal interphalangeal joints, accompanied by morning stiffness lasting more than one hour and intermittent episodes of enthesitis of the Achilles tendons, lasting a few weeks and self-resolving, recurring over the previous two years. During a prior emergency department visit, he had been started on 7.5 mg of prednisolone per day for symptomatic control of inflammatory polyarthritis, with no clinical suspicion of septic arthritis and inflammatory markers unavailable at that time.

Five weeks before the Internal Medicine clinic visit, he noticed progressive swelling and discomfort in the posterior aspect of the left knee, extending distally along the calf.

**Figure 1:**
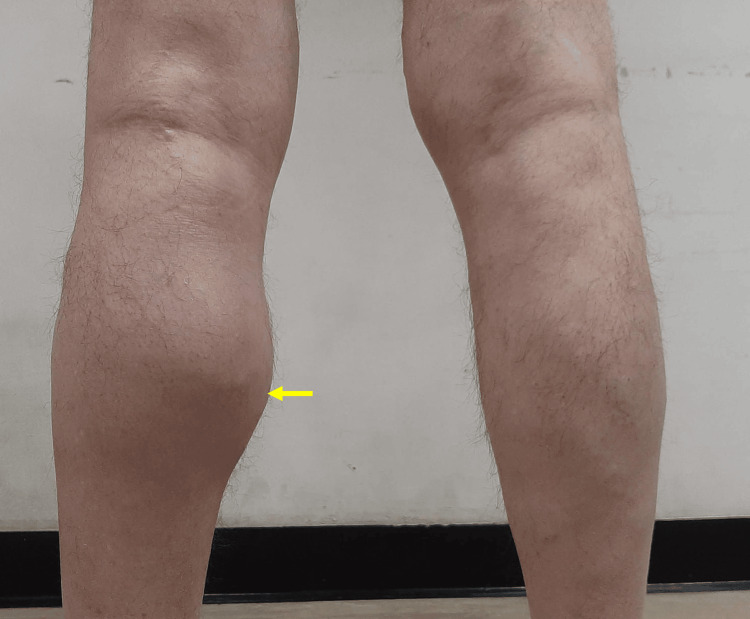
Posterior view of the legs showing a large popliteal mass. Posterior view of the lower extremities showing a fusiform enlargement of the proximal and mid-posterior left calf, resulting in noticeable asymmetry compared to the right side (yellow arrow). The finding is consistent with a large Baker’s cyst extending distally from the popliteal fossa along the gastrocnemius–semimembranosus interval.

On physical examination, there was visible distension of the left popliteal fossa with extension into the gastrocnemius region (Figure 1).

A fluctuant, non-tender mass was palpable, and knee flexion was mildly limited due to tension. No erythema, warmth, or signs of infection were noted. The neurovascular examination was unremarkable. There were no signs of active arthritis in both hands.

**Figure 2:**
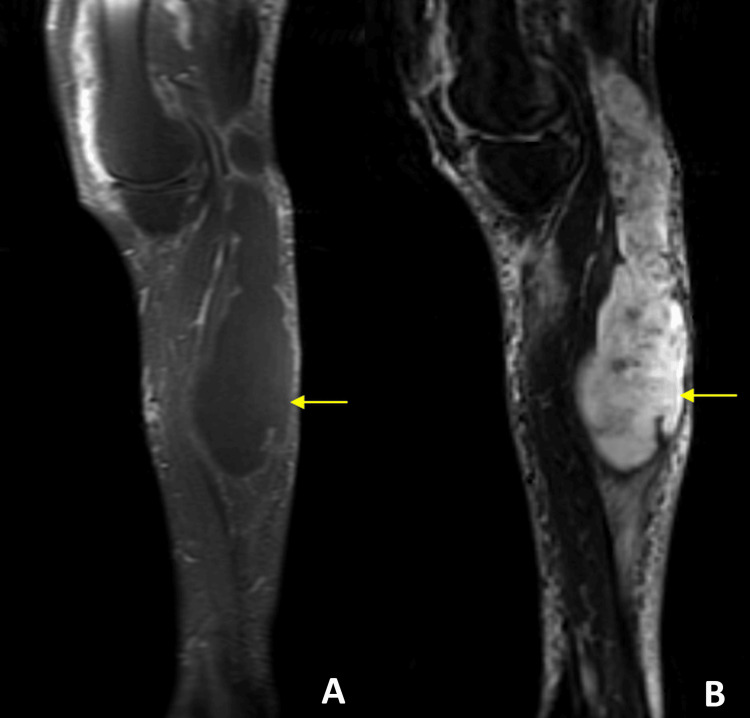
Sagittal MRI demonstrating a giant Baker’s cyst (yellow arrows) with mass effect. (A) The sagittal T1-weighted post-contrast image shows the cyst as a well-defined lesion with relatively homogeneous signal intensity. (B) The sagittal STIR image demonstrates the cyst with characteristic hyperintense signal consistent with fluid content. STIR: Short Tau Inversion Recovery

**Figure 3:**
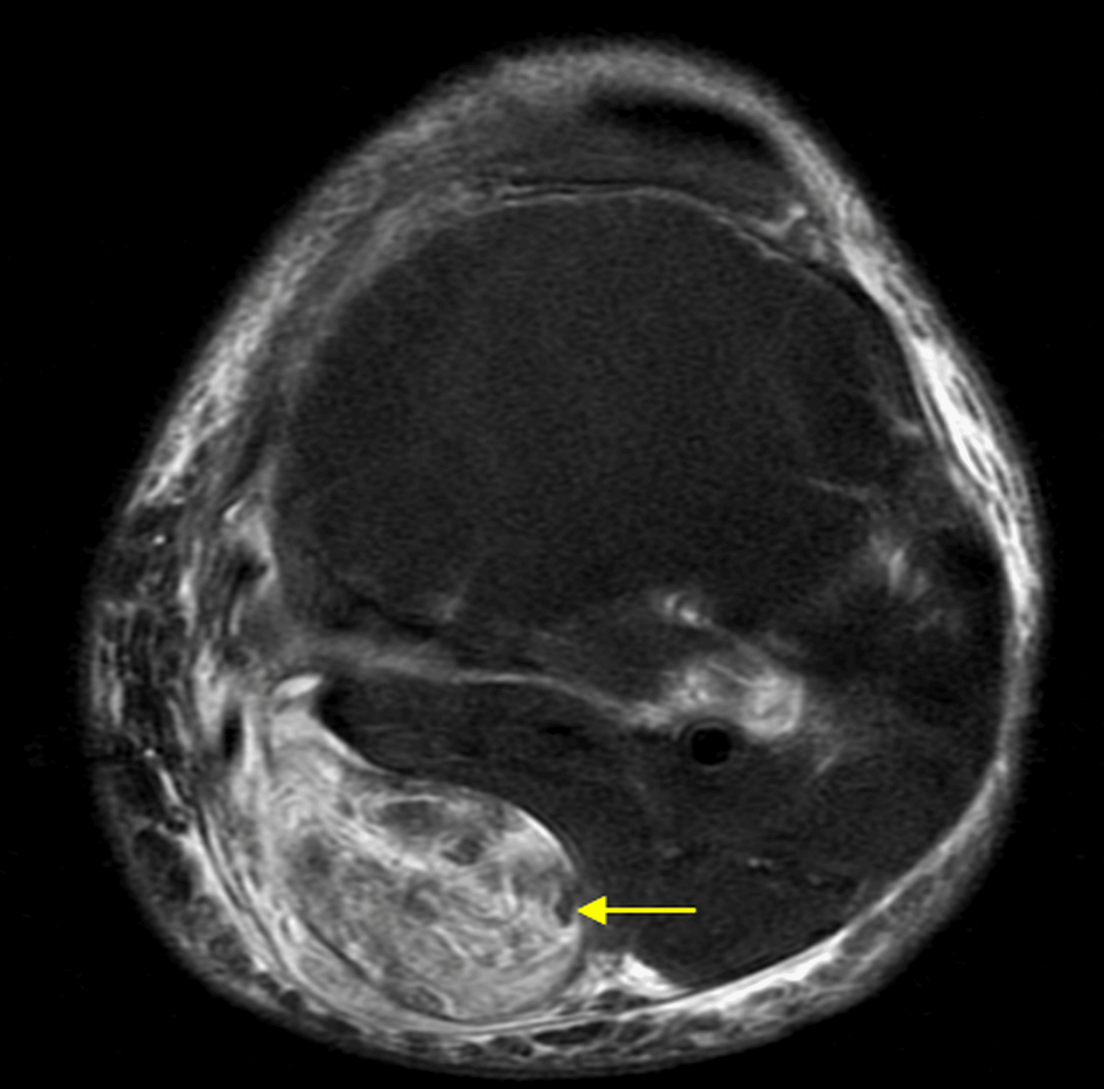
Axial MRI demonstrating a well-defined fluid-filled popliteal mass. The axial T2-weighted fat-suppressed image demonstrates a well-defined, hyperintense lesion (yellow arrow) with fluid signal intensity located in the popliteal fossa, exerting mild compression on the adjacent soft tissues.

Laboratory evaluation revealed a markedly elevated rheumatoid factor and anti-cyclic citrullinated peptide (anti-CCP) antibodies, confirming the diagnosis of RA. Magnetic resonance imaging (MRI) of the left knee demonstrated a large, well-defined Baker’s cyst measuring 24 cm in longitudinal length and 5 × 7 cm in transverse diameters (Figures 2, 3). The cyst exhibited heterogeneous signal intensity on all sequences, without synovial thickening, pannus formation, septation, or neoplastic features, findings consistent with post-hemorrhagic content. The left knee had a heterogeneous effusion with synovial thickening and diffuse edema of the peri-capsular soft tissues.

The patient continued corticosteroid therapy and began weekly methotrexate for the management of RA. He was subsequently evaluated by the orthopedic team, who performed surgical drainage of the cyst. This approach was chosen because the cyst’s exceptional size, its significant mass effect, and the heterogeneous post-hemorrhagic content seen on MRI made ultrasound-guided aspiration less feasible and more likely to be incomplete as a first-line intervention. A sample of joint fluid was sent for bacteriological analysis, which returned negative, ruling out septic arthritis. The postoperative course was uneventful, with resolution of swelling and symptomatic improvement. However, several weeks later, the cyst recurred at a smaller size, and ultrasound-guided aspiration with corticosteroid injection was performed, leading to remission of the cyst. Systemic treatment with methotrexate, and later etanercept, resulted in improved control of RA, and no further recurrence of the Baker’s cyst was observed thereafter. Presently our patient is under weekly methotrexate (20mg po) and etanercept (50mg sc), asymptomatic and with no signs of the giant Baker’s cyst.

## Discussion

Baker’s cysts are a common finding in RA due to chronic synovitis and increased intra-articular pressure [2]. Most are small and asymptomatic, but in rare cases they can reach remarkable dimensions, as in our patient with a 24-cm cyst.

The main clinical challenge lies in diagnosis. A popliteal mass may mimic several conditions, including deep vein thrombosis, popliteal artery aneurysm, hematoma, or soft-tissue tumors. Rupture of a cyst can present as acute calf pain and swelling, producing the so-called pseudothrombophlebitis syndrome [5]. Clinical examination alone is often insufficient, particularly in giant cysts. Imaging is therefore essential: ultrasound is useful as a first-line tool, while magnetic resonance imaging (MRI) provides detailed information about cyst size, internal content, and its relationship with adjacent structures [6,7]. In our case, MRI demonstrated a well-defined cyst with heterogeneous content, excluded neoplastic or pannus components, and revealed a mass effect on the medial gastrocnemius without evidence of rupture.

Giant Baker’s cysts are rarely described in the literature, with most reported lesions measuring only a few centimeters and only isolated case reports documenting cysts exceeding 20 cm in length. Similar to our case, these very large cysts often show heterogeneous or hemorrhagic internal content on imaging and may cause substantial mass effect on adjacent muscles or neurovascular structures. Previous reports indicate that aspiration with corticosteroid injection, although frequently attempted, is associated with a higher recurrence rate in large cysts, and surgical drainage is often required when symptoms are significant or mass effect is present. As described in the literature, long-term control of the underlying inflammatory disease, particularly RA, is essential to reduce the likelihood of recurrence and to achieve sustained remission [2,4].

Management should be individualized. Small, asymptomatic cysts can be managed conservatively with observation, physical therapy, and optimization of the underlying joint disease. Aspiration and corticosteroid injection may provide temporary relief in symptomatic cysts, although recurrence is common [2,8]. Surgical drainage or excision is generally reserved for large, symptomatic, or complicated cysts, particularly when there is neurovascular compression or suspicion of rupture [9]. In patients with RA, long-term control of synovial inflammation is crucial to prevent recurrence [4].

Our case illustrates the rare occurrence of a giant Baker’s cyst associated with RA, underscores the pivotal role of MRI in confirming the diagnosis and excluding potential mimickers, and demonstrates that surgical drainage along with treatment of the RA can be an effective therapeutic approach when conservative measures are insufficient.

## Conclusions

Baker’s cysts are a common finding in patients with RA, but they rarely reach giant proportions. When they do, they can mimic other conditions and cause significant morbidity by compressing adjacent structures. This case underscores the importance of considering Baker’s cysts in the differential diagnosis of popliteal masses, particularly in patients with inflammatory joint disease, and highlights the crucial role of MRI in both diagnosis and surgical planning. Ultimately, effective management requires not only appropriate treatment of the cyst itself but also optimal control of the underlying RA to minimize the risk of recurrence.
